# The complete mitogenome of *Euschemon rafflesia* (Lepidoptera: Hesperiidae)

**DOI:** 10.1080/23802359.2017.1292478

**Published:** 2017-02-23

**Authors:** Jing Zhang, Qian Cong, Jinhui Shen, Xiao-Ling Fan, Min Wang, Nick V. Grishin

**Affiliations:** a Departments of Biophysics and Biochemistry, University of Texas Southwestern Medical Center, Dallas, TX, USA;; b Department of Entomology, South China Agricultural University, Guangzhou, China;; c Howard Hughes Medical Institute, University of Texas Southwestern Medical Center, Dallas, TX, USA

**Keywords:** Next-generation sequencing, phylogeny, Euschemoninae, frenulum, Coeliadinae

## Abstract

We assembled a complete mitochondrial genome of a unique Australian skipper butterfly *Euschemon rafflesia* (Hesperiidae) from next generation sequencing reads. The 15,447 bp mitogenome covers 13 protein-coding genes (PCGs), 22 transfer RNA genes (tRNAs), 2 ribosomal RNA genes (rRNAs), and an A + T-rich region. Its gene order is typical for mitogenomes of Lepidoptera. Phylogenetic analysis places *Euschemon rafflesia* as a sister to the rest of Hesperiidae except Coeliadinae.

The Regent Skipper (*Euschemon rafflesia*) is an Australian endemic and the only member in the subfamily Euschemoninae (family Hesperiidae). It is a showy large butterfly with black, yellow-spotted wings and red abdomen tip, over 5 cm in wingspan (Figure 1). Like no other butterfly, it possesses a frenulum and retinaculum (in males only) (Braby 2000), frequently present in moths to couple the wings. Phylogenetic affinities of *Euschemon* have been debated for years, and it has even been considered to be a moth by earlier authors (Watson 1893). Later, it has been placed in or near Hesperiidae, possibly related to *Celaenorrhinus* (Braby 2000). Recently, *Euschemon* was given a subfamily status based on the combination of DNA and morphological evidence (Warren et al. 2008; Warren et al. 2009), the result corroborated in an expanded phylogenetic study (Sahoo et al. 2016).

**Figure 1.**
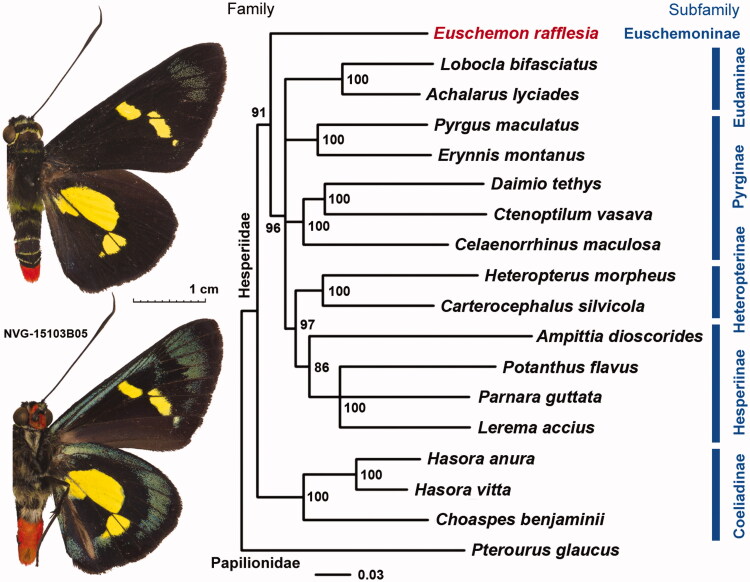
Maximum likelihood tree of complete mitogenomes of 17 Hesperiidae species rooted with *Pterourus glaucus* (Papilionidae). *Euschemon rafflesia* with mitogenome reported here is shown first and the specimen sequenced (voucher NVG-15103B05) is pictured on the left, dorsal and ventral sides above and below, respectively. Numbers by the nodes show bootstrap support values and branches with bootstrap less than 60% are collapsed. GenBank accessions for sequences are: *Achalarus lyciades* NC_030602.1; *Ampittia dioscorides* KM102732.1; *Celaenorrhinus maculosa* NC_022853.1; *Daimio tethys* NC_024648.1; *Euschemon rafflesia* KY513288; *Erynnis montanus* NC_021427.1; *Hasora anura* NC_027263.1; *Hasora vitta* NC_027170.1; *Heteropterus morpheus* NC_028506.1; *Choaspes benjaminii* NC_024647.1; *Lerema accius* NC_029826.1; *Lobocla bifasciatus* NC_024649.1; *Carterocephalus silvicola* NC_024646.1; *Potanthus flavus* NC_024650.1; *Parnara guttata* NC_029136.1; *Pyrgus maculatus* NC_030192.1; *Ctenoptilum vasava* NC_016704.1; *Papilio glaucus* NC_027252.

To better understand the phylogeny of Hesperiidae and clarify the phylogenetic position of the Regent Skipper, we sequenced, assembled and annotated the complete mitogenome of *Euschemon rafflesia rafflesia* from the male voucher NVG-15103B05 in the National Museum of Natural History collection (Smithsonian Institution, Washington DC) from Australia: Queensland, Southbrook, collected around 1946. The specimen is illustrated in Figure 1. A single leg was used for DNA extraction. Methods for genomic DNA extraction, library construction, next-generation sequencing, and computational procedures have been reported by us previously (Shen et al. 2015; Cong & Grishin 2016; Cong et al. 2016a, 2016b; Shen et al. 2016). The mitogenome of *Lobocla bifasciatus* (Kim et al. 2014) was used as a reference to search for (‘bait’) similar sequence reads using BWA (Li & Durbin 2009). Nearly 0.8% (159,718 out of 20,472,582) of *E. rafflesia* total genomic reads were extracted by BWA for mitogenome assembly (Hahn et al. 2013). The complete mitogenome of *E. rafflesia* was assembled *de novo* using Platanus (Kajitani et al. 2014) followed by a manual gap-closing procedure.

The complete mitogenome of *Euschemon rafflesia* is 15,447 bp in length (Genbank: KY513288) and is AT-rich, with a base composition of 39.1% A, 42.3% T, 7.4% G, and 11.2% C. It retains the typical insect mitogenome gene set, including 13 PCGs (ND1-6, COX1-3, ND4L, ATP8, ATP6, and CYTB), 22 tRNA genes (two for serine and leucine and one for each of the rest amino acids), 2 ribosomal RNAs (rrnL and rrnS), and an A + T-rich D-loop control region. As in many Lepidoptera mitogenomes, the exact start of COX1 gene is unknown, but is probably the codon TTG (Kim et al. 2009). The typical start codon ATN is used in other genes. COX1, COX2, ND4 and ND5 genes have an incomplete stop codon T, and a complete TAA codon is likely formed during mRNA maturation (Ojala et al. 1981; Boore 1999). The length of tRNAs ranges from 59 to 71 bp. The size of the two rRNAs are 1351 and 775 bp, respectively. A 453 bp A + T-rich region connects rrnS and tRNA-Met.

To phylogenetically place *Euschemon rafflesia* within Hesperiidae with available mitogenomes (Hao et al. 2012; Kim et al. 2014; Shen et al. 2015; Shao et al. 2015; Wang et al. 2013, 2014, 2015; Shen et al. 2016; Cong & Grishin 2016), we constructed RaxML (Stamatakis, 2006) maximum likelihood tree rooted with *Pterourus glaucus* (Papilionidae) mitogenome (Shen et al. 2015) (Figure 1). The placement of *Euschemon* as a sister to the rest of Hesperiidae except Coeliadinae is strongly supported and is in agreement with the previous results (Warren et al. 2008, 2009; Sahoo et al. 2016). The tree topology is consistent with previous phylogenetic studies (Warren et al. 2008, 2009; Sahoo et al. 2016): Coeliadinae are the sister to all other Hesperiidae; relationship between Eudaminae and Pyrginae is unresolved; Heteropterinae are the sister to Hesperiinae within which Aeromachini (represented by *Ampittia*) is the sister to the rest. As seen previously (Shen et al. 2016), bootstrap on mitogenomes is insufficient to support monophyly of Pyrginae, and the topology within the crown Hesperiinae group needs to be investigated further. In conclusion, the complete mitogenome of *Euschemon rafflesia* clarifies its phylogenetic position and strengthens the evidence for this unique skipper to be treated as a sole representative of the subfamily Euschemoninae.
